# Dendritic cell immunoreceptor 1 alters neutrophil responses in the development of experimental colitis

**DOI:** 10.1186/s12865-015-0129-5

**Published:** 2015-10-23

**Authors:** Sumika Tokieda, Marie Komori, Toshifumi Ishiguro, Yoichiro Iwakura, Kazuhiko Takahara, Kayo Inaba

**Affiliations:** Laboratory of Immunobiology, Department of Animal Development and Physiology, Division of Systemic Life Science, Graduate School of Biostudies, Kyoto University, Yoshida-Konoe, Sakyo, Kyoto, 606-8501 Japan; Core Research for Evolutional Science and Technology (CREST), Japan Science and Technology Agency (JST), Tokyo, Japan; Laboratory of Molecular Pathogenesis, Center for Experimental Medicine and Systems Biology, Institute of Medical Science, University of Tokyo, 4-6-1 Shirokanedai, Minato-ku, Tokyo 108-8639 Japan; Present addresses: Research Institute for Biomedical Sciences, Tokyo University of Science, Yamasaki 2641, Noda, Chiba, 278-0022 Japan

**Keywords:** C-type lectin receptor, DCIR, DSS colitis, Myeloperoxidase, Neutrophil

## Abstract

**Background:**

Ulcerative colitis, an inflammatory bowel disease, is associated with the massive infiltration of neutrophils. Although the initial infiltration of neutrophils is beneficial for killing bacteria, it is presumed that persistent infiltration causes tissue damage by releasing antibacterial products as well as inflammatory cytokines. A murine C-type lectin receptor, dendritic cell immunoreceptor 1 (Dcir1), is expressed on CD11b^+^ myeloid cells, such as macrophages, dendritic cells and neutrophils. It was reported that Dcir1 is required to maintain homeostasis of the immune system to prevent autoimmunity, but it is also involved in the development of infectious disease resulting in the enhanced severity of cerebral malaria. However, the role of Dcir1 in intestinal immune responses during colitis remains unclear. In this study, we investigated the role of Dcir1 in intestinal inflammation using an experimental colitis model induced with dextran sodium sulfate (DSS).

**Results:**

In contrast to wild type (WT) mice, *Dcir1*^*−/−*^ mice exhibited mild body weight loss during the course of DSS colitis accompanied by reduced colonic inflammation. Dcir1 deficiency caused a reduced accumulation of neutrophils in the inflamed colon on day 5 of DSS colitis compared with WT mice. Consistently, the production of a neutrophil-attracting chemokine, MIP-2, was also decreased in the *Dcir1*^*−/−*^ colon compared with the WT colon on day 5. There were fewer myeloperoxidase-positive neutrophils in the inflamed colon of *Dcir1*^*−/−*^ mice than in that of WT mice. Moreover, bone marrow neutrophils from *Dcir1*^*−/−*^ mice produced less reactive oxygen species (ROS) by lipopolysaccharide stimulation than those from WT mice. This suggests that Dcir1 deficiency decreases the accumulation of tissue destructive neutrophils during DSS colitis.

**Conclusion:**

Dcir1 enhances the pathogenesis of DSS colitis by altering neutrophil recruitment and their functions.

## Background

Inflammatory bowel disease (IBD) is an inflammatory disorder of the gastrointestinal tract and is clinically subdivided into two forms: ulcerative colitis (UC) and Crohn’s disease [1]. The disease is characterized by inflammation of the intestine, and the clinical symptoms include weight loss, diarrhea accompanied by blood, and abdominal pain [2, 3]. The affected areas typically show transmural inflammation characterized by lymphoid hyperplasia, submucosal edema, ulcerative lesions and fibrosis [2]. The etiology of IBD still remains largely unclear. In recent years, however, epidemiologic and genetic studies in human and IBD-related animal models have suggested that genetic susceptibility factors affecting the host immune system and environmental factors such as gut microbiota contribute to the initiation and chronicity of IBD [4–6].

Neutrophils produce massive amounts of reactive oxygen species (ROS) that effectively destroy pathogens [7] and numerous antibacterial intracellular granules that contain myeloperoxidase (MPO) among other molecules [8]. Hypochlorous acid and other chlorinated oxidants generated by a catalytic reaction between chloride and hydrogen peroxide by MPO result in tissue injury under inflammatory conditions and protect from pathogenic infection [8–10]. In the gut of IBD patients, the excessive recruitment and accumulation of activated neutrophils were observed in association with mucosal injury and debilitating disease symptoms [7]. IBD can be accelerated by innate immune cells, which recognize pathogen-associated and damage-associated molecular patterns through pattern recognition receptors (PRRs) [11, 12].

Dendritic cell (DC) immunoreceptor (DCIR), a C-type lectin receptor, is a PRR and is expressed on DCs, monocytes, macrophages, B cells, and neutrophils in humans [13, 14]. The cytoplasmic tail of DCIR contains an immunoreceptor tyrosine-based inhibitory motif (ITIM), which transduces immunoregulatory signals *via* interactions with Src homology 2 domain tyrosine phosphatase (SHP)-1 and SHP-2 [14]. Similar to other C-type lectin receptors such as Mincle, which is expressed on macrophages and recognizes pathogen-associated molecular patterns and damage-associated molecular patterns to initiate an immune response [15], DCIR can bind to endogenous carbohydrates as well as pathogenic and viral antigens [16, 17].

In mice, Dcir consists of four homologs (Dcir1 to 4). Although Dcir1 and Dcir2 contain ITIM sequences, only Dcir1 has been shown to interact with SHP-1 and SHP-2 [14, 18]. Several studies showed that murine Dcir1 is also expressed on CD11b^+^ myeloid cells, including monocytes, macrophages, and DCs; however, Dcir2 is mainly expressed on CD8^−^ DCs [14, 16, 19]. We recently reported that a lack of Dcir1 exacerbated autoimmune arthritis caused by the spontaneous expansion and activation of DCs [20]. Other studies demonstrated that Dcir1 was essential for the development of cerebral malaria and that Dcir1 deficiency reduced brain inflammation accompanied with decreased tumor necrosis factor (TNF)-α production and modulated the activation of T cells [21]. These results suggest that Dcir1 is involved in disease symptoms both positively and negatively.

Infiltration by neutrophils and monocytes is a characteristic histologic feature of lesions of active UC [22]. Neutrophils and Ly6C^hi^ monocytes are considered to promote intestinal inflammation during acute dextran sulfate sodium (DSS) induced colitis (DSS colitis) [23, 24]; therefore, we hypothesized that Dcir1 may have an effect on the symptoms of colitis by altering the behavior and functions of these cells.

This study examined the effect of Dcir1 deficiency in intestinal inflammation using a DSS colitis model. We found that *Dcir1*^*−/−*^ mice developed milder colitis with low accumulation of activated neutrophils and expression of macrophage inflammatory protein-2 (MIP-2) in the colon than WT mice. Neutrophils from *Dcir1*^*−/−*^ bone marrow also had less ROS production in response to lipopolysaccharide (LPS) than those from WT mice. In conclusion, our data suggest that Dcir1 is responsible for the development of acute colitis by promoting neutrophil recruitment and altering neutrophil functions.

## Methods

### Mice

C57BL/6 mice were purchased from Japan SLC (Hamamatsu, Japan). Dcir1-deficient (*Dcir1*^*−/−*^) mice on a C57BL/6 background [20] were obtained from Dr. Iwakura (Tokyo University of Science, Chiba, Japan). All mice used in this study were female and aged between 8 and 10 weeks. *Dcir1*^*−/−*^ or C57BL/6 wild type (WT) control mice were co-housed after weaning until use. All experiments were performed according to the Institutional Guidelines for Animal Use and Experimentation of Kyoto University (Permit Number: Lif-K13020).

### DSS colitis

*Dcir1*^*−/−*^ or WT mice were provided with 2.0 % (wt/vol) DSS (reagent grade DSS salt; molecular mass = 36–50 kDa) (MP Biomedicals, Irvine, CA) dissolved in drinking water for 5 days, followed by 3 days with normal water. Mice given normal water for the entire period were used as controls. Individual mouse body weight was recorded daily for each group. On day 8, the length of the cecum and colon was measured after dissection by placing them on a paper towel.

### Histological analysis

After washing in phosphate buffered saline (PBS), the cecum and colon were cut longitudinally and fixed with Bouin’s solution (71.4 % saturated picric acid, 23.8 % formaldehyde, and 4.8 % acetic acid) for 2 h at room temperature and embedded in paraffin. Two sections (4–6 μm thick) from a remote area of the cecum, and sections from the descending, transverse, and ascending colon from individual mice were stained with hematoxylin-eosin (HE) and observed using a BZ-8000 Biozero imaging device (Keyence, Osaka, Japan).

Histological colitis scores were determined as previously described [25]. Briefly, subscores were as follows: **ulceration**: 0, no ulcers; 1, one ulcer; 2, two ulcers; 3, three ulcers; and 4, > 3 ulcers: **epithelium**: 0, normal morphology; 1, loss of goblet cells; 2, loss of goblet cells in large areas; 3, loss of crypts; and 4, loss of crypts in large areas (>50 %) and/or foci of polypoid regeneration: **infiltration**: 0, no infiltrate; 1, infiltrate around crypt bases; 2, infiltrate reaching to lamina muscularis mucosae; 3, extensive infiltration reaching lamina muscularis mucosae, thickening of the mucosa with abundant edema; and 4, infiltration of the lamina submucosa: **lymphoid follicles**: 0, no lymphoid follicles; 1, one lymphoid follicle; 2, two lymphoid follicles; 3, three lymphoid follicles; and 4, > 3 lymphoid follicles. The colitis score of each mouse represents the mean of the different histological subscores.

For the immunohistological detection of neutrophils, 10-μm-thick frozen sections from DSS-treated WT and *Dcir1*^*−/−*^ colonic tissues were fixed with 4 % paraformaldehyde, and stained with rabbit anti-MPO polyclonal antibody (Ab) (Abcam, Cambridge, MA) followed by incubation with donkey-anti rabbit IgG-Cy3 (BioLegend, San Diego, CA) and then with anti-Ly6G-FITC (BD Biosciences, San Jose, CA), anti-CD11b-APC (eBioscience, San Diego, CA) and DAPI (Wako Pure Chemical Industries, Ltd., Osaka, Japan). After refixation with 1 % paraformaldehyde for 10 min, the specimens were mounted with Vectashield mounting medium (Vector Laboratories, Burlingame, CA) and observed using a deconvolution microscope BX51-FL (Olympus, Tokyo, Japan). MPO^+^CD11b^+^Ly6G^+^ cells were counted as activated neutrophils. Data represent number of neutrophils/whole area of a vertical section.

### Isolation of mononuclear cells

Isolation of lamina propria mononuclear cells (LPMCs) was performed as described previously [26] with some modifications. Briefly, colons were opened longitudinally and washed to remove fecal content by shaking in Hank’s Balanced Salt Solution containing 5 mM EDTA for 20 min at 37 °C, followed by the removal of epithelial cells and fat tissue. After cutting the colon into small pieces, tissues were incubated with RPMI 1640 containing 4 % fetal bovine serum (FBS), 1 mg/ml collagenase D (Roche, Basel, Switzerland), 0.5 mg/ml dispase (Gibco, Carlsbad, CA) and 40 μg/ml DNase (Sigma–Aldrich, St. Louis, MO) for 50 min at 37 °C in a shaking water bath. The digested tissues were washed with Hank’s Balanced Salt Solution containing 5 mM EDTA, resuspended in 4 ml of 40 % Percoll (GE Healthcare, Tokyo, Japan) and overlaid on 2 ml of 80 % Percoll in a 15 ml conical tube. Percoll gradient separation was performed by centrifugation at 1200 × *g* for 20 min at 25 °C. The cells at the interface were collected and used as LPMCs.

### Flow cytometry

LPMCs suspended in staining buffer (PBS containing 1 % FBS, 5 mM EDTA and 0.02 % NaN_3_) were incubated with anti-CD16/32 monoclonal antibodies (mAb) for 30 min at 4 °C to block nonspecific binding. Then, cells were stained for 30 min at 4 °C with a combination of the following fluorescent mAbs: FITC-conjugated anti-Ly6G or anti-DX5 (BD Biosciences), PE-conjugated anti-CD11c (BD Biosciences), PE-Cy7-conjugated anti-CD11b (BD Biosciences), Alexa Fluor 647-conjugated anti-Ly6C (BioLegend), APC-conjugated anti-CD3ε (eBioscience) or anti-CD19 (eBioscience). Dead cells were gated out by staining with 7-aminoactinomycin D (7-AAD) (Life Technologies, Carlsbad, CA). Stained cells were acquired using FACSCalibur (BD Biosciences) or FACSAriaIII (BD Biosciences), and data were analyzed with FlowJo software (TreeStar Inc., Ashland, OR).

### Cytokine measurement

After treatment with DSS for 5 days, ceca and colons were obtained from *Dcir1*^*−/−*^ or WT mice. Colons were divided into three pieces and ceca were washed and weighed. Each segment was cultured in RPMI 1640 medium for 24 h, and cytokines, interleukin (IL)-1β, IL-6, IL-10, IL-12, TNF-α and monocyte chemoattractant protein-1 (MCP-1), in the medium were measured using a cytometric bead array (BD Biosciences) according to the manufacturer’s instructions. Data were analyzed with FCAP Array software (BD Biosciences). Data were analyzed by FCAP Array software (BD Biosciences). The concentrations of MIP-2 and KC were measured by ELISA (PeproTech, Rocky Hill, NJ) according to the manufacturer’s instructions.

### Migration assay of neutrophils

Chemotactic activity of neutrophils was assessed using 3-μm pore size transwells (Costar, Washington, DC). After removing red blood cells with ACK lysis buffer (0.15 M NH_4_Cl, 10 mM KHCO_3_, 0.1 mM Na_2_EDTA, pH 7.2), bone marrow cells were blocked with anti-CD16/32 mAb, and stained with FITC-anti-Ly6G and PE-anti-CD11b mAbs (eBioscience). Then, cell suspensions (10^6^/well) in RPMI 1640 containing 0.1 % bovine serum albumin in the upper well were placed above a lower well with or without 100 ng of MIP-2 (PeproTech). One hour later, input cells and cells that had migrated into the lower well were counted and analyzed by flow cytometry.

### Neutrophil preparation and measurement of oxidative burst

Ly6G^+^ CD11b^+^ cells in bone marrow were sorted as neutrophils using FACSAriaIII. Purified neutrophils were stimulated in the presence or absence of 100 ng/ml Ultra-pure LPS from *E. coli* O111:B4 (InvivoGen, San Diego, CA) or 10 ng/ml granulocyte-macrophage colony-stimulating factor (GM-CSF) (Kirin Brewery, Gunma, Japan) for 6 h, and then incubated with 10 μM of dihydrorhodamine-123 (DHR-123) (Sigma–Aldrich) for 30 min in quadruple cultures. Fluorescence intensity was determined using FACSCalibur. Results were calculated according to the following formula: rhodamin-123 fluorescence = mean fluorescence intensity (MFI) of stimulated cells/MFI of unstimulated cells × 100.

### Statistical analysis

Differences between *Dcir1*^*−/−*^ and WT groups or treated and nontreated groups were evaluated using the unpaired Student’s *t*-test. A *P*-value of < 0.05 was considered statistically significant.

## Results

### Milder symptoms of DSS colitis in *Dcir1*^*−/−*^ compared with WT mice

**Fig. 1:**
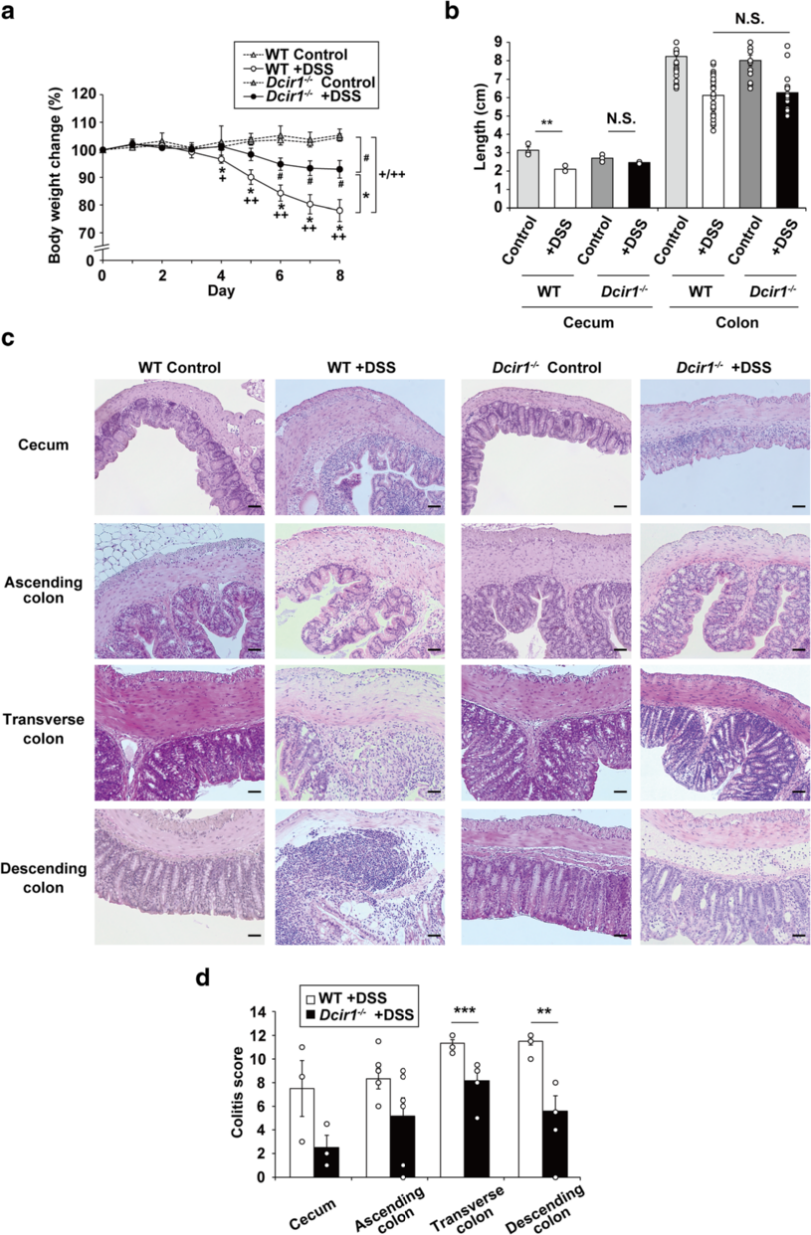
Decreased susceptibility of *Dcir1* ^*−/−*^ mice to DSS colitis than WT mice. **a** Body weight change of WT and *Dcir1* ^*−/−*^ mice during DSS colitis is shown. Data are representative of three independent experiments (*n* = 4–7 mice/group). *^, #, +^ *P* < 0.05, ^++^ *P* < 0.001. **b** Length of cecum and colon on day 8 was measured. Cecum and colon data are representative of two (*n* = 3) and six (*n* = 15–37) independent experiments, respectively. **P < 0.01, ***P < 0.005. **c** Cross-sections of cecum on day 8 were observed after hematoxylin-eosin staining (magnification × 20). Scale bars represent 50 μm. **d** Histological colitis scores of various parts of intestine on day 8 were indicated. Data are representative of two independent experiments (*n* = 3 for cecum and *n* = 6 for colon). **P < 0.01, *** *P* < 0.005. Data are expressed as the mean ± SEM

To elucidate the roles of Dcir1 in acute colitis, *Dcir1*^*−/−*^ and WT mice were treated with drinking water containing 2.0 % DSS for 5 days, followed by normal water for 3 days. DSS-treated WT and *Dcir1*^*−/−*^ mice started losing body weight on day 4 and day 5, respectively, and both groups showed a gradual loss of weight thereafter (Fig. 1a). Weight loss in DSS-treated WT mice was significantly greater than that in *Dcir1*^*−/−*^ mice from day 4, but there was no significant difference in the colon length of both groups (Fig. 1a, b).

Damage caused by ulceration and submucosal inflammation was identified by disruption of the epithelial barrier [27]. Histological analysis of the colons from untreated *Dcir1*^*−/−*^ mice showed intact epithelium, absence of edema, and normal muscle architecture (Fig. 1c). However, on day 8 after the induction of colitis, *Dcir1*^*−/−*^ mice showed milder colon tissue damage than WT mice, characterized by mucosal architecture, thickening of smooth muscle, presence of crypt abscesses, and cellular infiltration (Fig. 1c), particularly in the transverse and descending colon (Fig. 1c, the third and bottom row). In the cecum, increased mucosal erosion was observed in both groups, but inflammatory cell infiltration was greater in WT mice than in *Dcir1*^*−/−*^ mice (Fig. 1c, top row). The colitis scores of cecum and colon sections also supported the macroscopic observations (Fig. 1d). These data suggest that Dcir1 accelerates the development and/or promotes acute colitis.

### Decreased infiltration of neutrophils during DSS colitis in *Dcir1*^*−/−*^ mice compared with WT mice

**Fig. 2:**
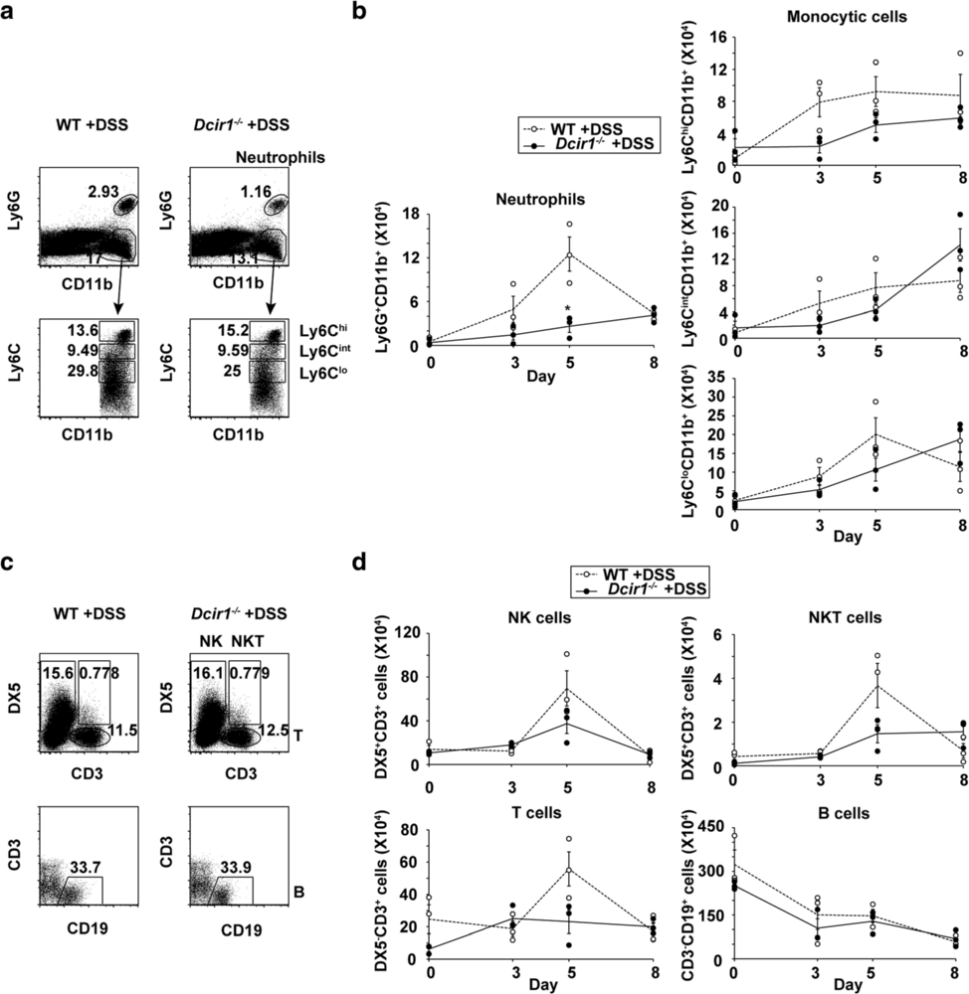
Decreased infiltration of neutrophils in *Dcir1* ^*−/−*^ mice intestine during DSS colitis. **a** Neutrophils (CD11b^+^Ly6G^+^) and monocytic cells (CD11b^+^Ly6G^low^Ly6C^low, int, hi^) in the intestine on day 5 after induction of DSS colitis were analyzed by flow cytometry. The numbers in dot plots indicate the percentages of cells in the gated populations. Monocytic cells were separated into three populations dependent upon Ly6C expression. **b** Numbers of neutrophils and monocytic myeloid cells during DSS colitis are shown. The populations were identified as in (**a**) Results are representative of two independent experiments (*n* = 3). *P < 0.05. **c** T cells (DX5^−^CD3^+^), NKT cells (DX5^+^CD3^+^), NK cells (DX5^+^CD3^−^) and B cells (CD3^−^ CD19^+^) in the intestine on day 5 after induction of DSS colitis were analyzed by flow cytometry. **d** Numbers of T cells, NKT cells, NK cells and B cells during DSS colitis were depicted. The populations were identified as in (**c**). Results are representative of two independent experiments (*n* = 3). *P < 0.05. Data are expressed as the mean ± SEM

Massive infiltration of various types of leukocytes in the colon is often accompanied by the progression of DSS colitis [28]. In *Dcir1*^*−/−*^ mice, the infiltration of neutrophils into the colon was significantly lower than in WT mice (Fig. 2a), especially on day 5 (Fig. 2b). At the same time point, Ly6C^hi^, Ly6C^int^, and Ly6C^lo^ monocytic cells were slightly decreased in *Dcir1*^*−/−*^mice compared with WT mice, although this was not statistically significant. Similarly, lower numbers of natural killer (NK) cells, NKT cells and T cells, but not B cells, infiltrated the colon in *Dcir1*^*−/−*^mice compared with WT mice on day 5, but this was not statistically significant (Fig. 2c and d). Although both immuno-suppressive and -activating subtypes were present in the infiltrating cells, it is known that neutrophils are the first effector cells observed in the colon during the onset of DSS colitis. Collectively, these findings suggest that, in the Dcir1 expressing cells, a decrease of neutrophils rather than of monocytes, contributes to the amelioration of DSS colitis in *Dcir1*^*−/−*^ mice.

### Decreased MIP-2 production in the intestine of *Dcir1*^*−/−*^ mice

**Fig. 3:**
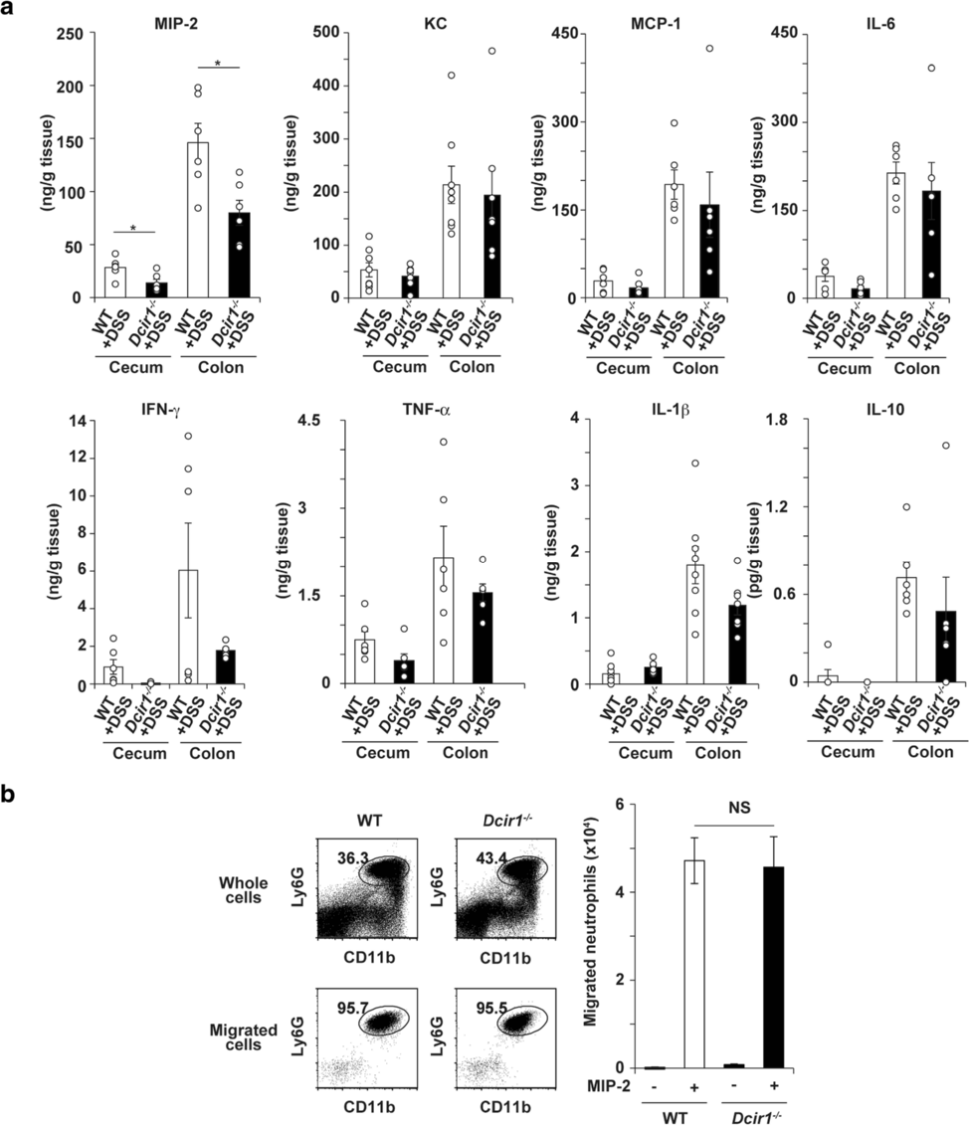
Decreased MIP-2 production and normal neutrophil responses in *Dcir1* ^*−/−*^ mouse colitis colon. **a** Chemokine and cytokine production in organ cultures was analyzed. Tissue fragments of ceca and colons from 5 days DSS-treated mice were cultured for 24 h, and chemokine and cytokine production was measured by CBA or ELISA. Data are the combined results of two independent experiments (*n* = 6). *P < 0.05. **b** Chemotactic activity of neutrophils to MIP-2 was assessed using transwells. After staining with FITC-anti-Ly6G and PE-anti-CD11b, bone marrow cells that migrated into the lower wells in the presence of 100 ng of MIP-2 were analyzed by flow cytometry after 1 h (*left panels*). Data are representative of two independent experiments (*n* = 4) (*right bar panel*). Data are expressed as the mean ± SEM

We next checked cytokine/chemokine production in *Dcir1*^*−/−*^ and WT mouse intestines using a whole tissue culture method [29, 30]. Notably, on day 5 of DSS treatment, neutrophil chemoattractant MIP-2, not but KC, production in the colon and cecum of *Dcir1*^*−/−*^ mice was significantly lower than in WT mice, consistent with the reduced accumulation of neutrophils in the *Dcir1*^*−/−*^ colons (Figs. 2b, 3a). Although the production of monocyte chemoattractant MCP-1 and several proinflammatory cytokines, such as IFN-γ and IL-1β, tended to be lower in *Dcir1*^*−/−*^ colons, the difference was not statistically significant (Fig. 3a). The production of other cytokines was not different between the WT and *Dcir1*^*−/−*^ colons. We could not detect IL-12 expression. These data collectively suggest that Dcir1 may accelerate neutrophil recruitment through the enhanced MIP-2 production in inflamed colons.

To exclude the possibility that *Dcir1*^*−/−*^ neutrophils are less competent to respond to MIP-2, the chemotactic activity of bone marrow neutrophils was assessed using a transwell culture system. Results showed that a lack of Dcir1 did not affect the migration capacity of neutrophils in response to MIP-2 (Fig. 3b).

### Decreased numbers of MPO^+^ neutrophils in the inflamed colon of *Dcir1*^*−/−*^ mice

**Fig. 4:**
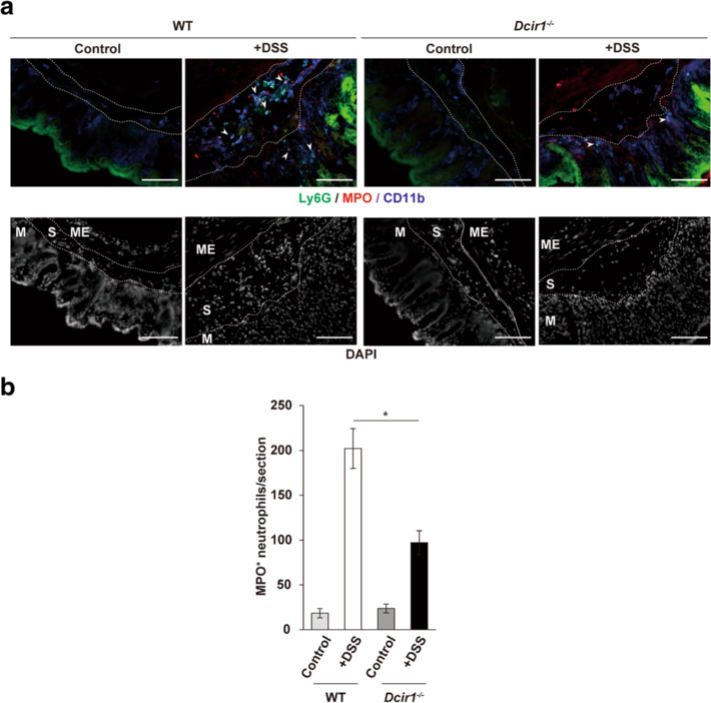
Decreased numbers of MPO^+^ neutrophils in *Dcir1* ^*−/−*^ mice colitic colon. **a** The descending colons from WT and *Dcir1* ^*−/−*^ mice that were untreated or treated with DSS for 5 days were stained with FITC-anti-Ly6G (green), anti-MPO (red) and APC-anti-CD11b (blue). Cell nuclei were visualized using DAPI (white). *Arrowheads* represent cells stained with triple colors. ME: muscle externa, S: submucosa, M: mucosa. Each area is delineated by a white dotted line. Magnification × 10. Scale bars represent 100 μm. **b** Numbers of MPO^+^ neutrophils (CD11b^+^Ly6G^+^) were counted in vertical sections of colon from WT and *Dcir1* ^*−/−*^ mice treated as in (**a**) Data are the combined results of two independent experiments (*n* = 3-5). *P < 0.05. Data are expressed as the mean ± SEM

MPO in neutrophils is involved in the production of ROS that are involved in both host defense and tissue injury [9, 10]. To identify the localization and numbers of MPO^+^ neutrophils, we stained cryosections of the colon using anti-MPO Ab in combination with anti-Ly6G and -CD11b mAbs on day 5. MPO^+^ neutrophils (CD11b^+^Ly6G^+^) infiltrated into the submucosal and mucosal areas of the colon in colitis-induced WT mice and *Dcir1*^*−/−*^ mice (Fig. 4a, arrowheads). However, fewer MPO^+^ neutrophils were accumulated in *Dcir1*^*−/−*^ mice colon than in WT mice colon (Fig. 4b). Without DSS treatment, there was no difference in the numbers of MPO^+^ neutrophils in WT and *Dcir1*^*−/−*^ colons. These results suggest that Dcir1 in WT mice enhances the infiltration of activated neutrophils into the colon, which exacerbates colitis.

### Altered ROS production from Dcir1^−/−^ neutrophils in response to LPS stimulation *in vitro*

**Fig. 5:**
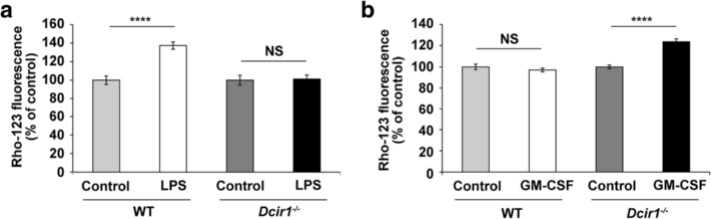
Involvement of Dcir1 in the oxidative burst of neutrophils in response to LPS and GM-CSF. **a** Neutrophils sorted from *Dcir1* ^*−/−*^ and WT bone marrow were stimulated with 100 ng/ml LPS for 6 h in the presence of DHR-123 for the last 30 min of culture, and conversion to rhodamine-123 (Rho-123) was assessed by flow cytometry. **b** ROS production of neutrophils was analyzed as in (**a**) after stimulation with 10 ng/ml GM-CSF for 6 h. Results are representative of two independent experiments (*n* = 4). ****P < 0.001. Data are expressed as the mean ± SEM

We next focused on ROS production by neutrophils lacking Dcir1. Neutrophils were purified from bone marrow and stimulated with LPS (Fig. 5a). In comparison with WT neutrophils, *Dcir1*^*−/−*^ neutrophils produced lower amounts of ROS in response to LPS. However, *Dcir1*^*−/−*^, but not WT, neutrophils produced more ROS in response to GM-CSF (Fig. 5b). This is consistent with a previous report that Dcir1 inhibited GM-CSF-dependent signaling [20]. These results suggest that Dcir1 enhances both infiltration of neutrophils into the inflamed colon, and their ability to produce ROS in response to microbes.

## Discussion

Here we demonstrated that Dcir1 is involved in acute intestinal inflammation caused by DSS. Of note, both *Dcir1*^*−/−*^ and WT mice demonstrated similar body weight changes in the early days of DSS intake; however, *Dcir1*^*−/−*^ mice showed attenuated body weight loss from day 4 and a better clinical score especially in the transverse and distal colon compared with WT mice on day 8. These results imply that Dcir1 enhances the progression of DSS colitis.

MIP-2 production was lower in colons from *Dcir1*^*−/−*^ mice on day 5 of DSS colitis, when the number of neutrophils reached a peak. MIP-2 expression is correlated with the clinical activity index in human UC and murine DSS colitis [31, 32], and its receptor, CXCR2, is a major mediator of neutrophil influx in some disease models including acute colitis [33, 34]. The depletion or blocking of CXCR2 was protective against acute and chronic DSS colitis [35, 36]. Similarly, high levels of CXCR1/2 and CXCR1/2 ligand expression were observed in the mucosa of UC patients [37]. Although we observed the normal migration of bone marrow *Dcir1*^*−/−*^ neutrophils toward MIP-2, we found reduced accumulation of MPO^+^ neutrophils in colons on day 5 of DSS colitis. Activated neutrophils secrete MPO, resulting in mucosal disruption and ulceration [7, 38, 39]. Therefore, our data suggest that Dcir1 contributes to the development of intestinal inflammation though the recruitment of neutrophils and their MPO production. Furthermore, bone marrow neutrophils from *Dcir1*^*−/−*^ mice produced smaller amounts of ROS compared with WT neutrophils in response to LPS. During colitis, the epithelial barrier is damaged, leading to interactions between commensal bacteria and neutrophils. In this situation, *Dcir1*^*−/−*^ neutrophils may produce small amounts of ROS compared with WT neutrophils. Commensal bacteria have been implicated in the pathogenesis of human IBD and several animal models including DSS colitis [40–42]. Therefore, a lack of Dcir1 might also contribute to ameliorating DSS colitis *via* the down modulation of ROS production.

We also found that a lack of Dcir1 enhanced GM-CSF signaling. Previous reports showed GM-CSF production in the colon of UC patients and DSS colitis-induced mice [43, 44]. The enhancement of GM-CSF signaling possibly suppresses colitis because *GM-CSF*^*−/−*^ mice are more susceptible to DSS colitis [45] and GM-CSF treatment results in significantly improved clinical parameters and histology of DSS colitis [46]. These results suggest that a lack of Dcir1 enhances GM-CSF signaling, resulting in an improvement of DSS colitis. Taken together, a lack of Dcir1 causes reduced neutrophil accumulation *via* downregulation of MIP-2 expression, reduced ROS production and enhanced GM-CSF signaling, which possibly leads to an improvement of DSS induced colitis. In contrast, it is known that ROS production increases direct bactericidal activity [47] and neutrophil extracellular traps (NET) formation [48] by neutrophils. Lack of Dcir1 might affect these neutrophil functions.

We previously reported that *Dcir1*^*−/−*^mice tended to develop autoimmune arthritis because of enhanced GM-CSF signaling and DCs development [20]. Furthermore, DCIR signaling induces the suppression of other pathways, such as B cell receptor signaling and proinflammatory cytokine production *via* TLR ligands [19, 49, 50]. However, Dcir1 enhances DSS colitis *via* several pathways as described here. Therefore, the balance between benefit and detriment caused by DCIR might be different in various types of disease models and experimental conditions. Recently, Hutter *et al*. reported no difference in DSS colitis symptoms observed between *Dcir1*^*−/−*^ and WT mice [51]. It was also reported that an increased cellular infiltration into the rectum of *Dcir1*^*−/−*^ mice was present at 7 days with 3 % DSS treatment. In our study, we used a different protocol (2.0 % DSS for 5 days followed by water for 3 days) for a more moderate induction of colitis, and observed an alleviation of body weight loss in *Dcir1*^*−/−*^ mice mainly after exchanging DSS for normal water. The simplest explanation for the different observations between studies is that the role of Dcir1 might be different depending on the symptoms of DSS colitis, or their use of stronger DSS colitis induction conditions compared with our study, which might have negated the benefit of a lack of Dcir1.

## Conclusions

We showed that a lack of Dcir1 suppressed the development of experimental DSS colitis. In the intestine of *Dcir1*^*−/−*^ mice with colitis, the number of tissue-injuring MPO^+^ neutrophils was lower than in WT mice, possibly because of the down-regulation of MIP-2 chemokine. Furthermore, bone marrow neutrophils produced reduced levels of ROS in response to LPS. Our results suggest that Dcir1 affects the accumulation and properties of neutrophils, and enhances the development of DSS colitis. Further studies regarding the mechanisms controlling neutrophils by DCIR will provide new clues to understand intestinal inflammation.

## Abbreviations

IBD: Inflammatory bowel disease
UC: Ulcerative colitis
ROS: Reactive oxygen species
MPO: Myeloperoxidase
DC: Dendritic cell
DCIR: DC immunoreceptor
ITIM: Immunoreceptor tyrosine–based inhibitory motif
SHP: Src homology 2 domain tyrosine phosphatase
DSS: Dextran sulfate sodium
MIP-2: Macrophage inflammatory protein-2
